# Can early treatment of patients with risk factors contribute to managing the COVID-19 pandemic?

**DOI:** 10.7189/jogh.10.010377

**Published:** 2020-06

**Authors:** Daniele Giammaria, Alessandro Pajewski

**Affiliations:** 1 Ophthalmology Department, Azienda “Ospedali Riuniti Marche Nord”, Pesaro, Italy; 2 Gran Sasso Science Institute (GSSI), L’Aquila, Italy

In recent months, the pandemic caused by Severe Acute Respiratory Syndrome-Coronavirus-2 (SARS-CoV-2) produced serious consequences in terms of both human lives lost and economic damage. For reasons still unclear, lethality rates reported for Coronavirus Disease-2019 (COVID-19) appear to differ significantly among countries [1]. Different health strategies for managing COVID-19 patients implemented in different countries may have played an important role in determining these lethality rates.

In most cases COVID-19 presents mild symptoms, and 50% of infected individuals are asymptomatic [2]. However, in about 20% of patients the disease causes severe clinical consequences that require hospitalization and, in some cases, intensive care [3]. The average time between the onset of symptoms and hospitalization is seven days [4]. These data confirm the clinical observation that patients with severe forms worsen to clinical conditions one week after the onset of symptoms, on average [5]. Individual factors influencing the natural history of the disease could cause such dissimilar clinical outcomes.

To date there are still no drugs officially approved for treatment of COVID-19. However, as already observed in SARS [6,7], it appears that early treatment with antiviral drugs could improve the natural history of COVID-19 [8,9]. Identifying in advance all subjects at risk of developing severe forms of COVID-19 and treating them with antivirals during early stages of infection could be one strategy to manage the pandemic.

COVID-19 appears to share several pathophysiological mechanisms with previous epidemics caused by SARS-CoV and Middle East Respiratory Syndrome (MERS)-CoV. As with SARS and MERS, the course of COVID-19 presents two phases: a first “viral” phase when cells are infected by the virus, the viral load increases rapidly and the host's immune response is triggered; and a second “immune” phase, which occurs in some patients only, when the immune system loses control of the inflammatory response, therefore causing serious damage to the lungs, kidneys and heart [10,11]. A common feature of coronaviruses is to replicate very quickly both in vitro and in vivo [12,13]. In SARS-CoV infections, high viral loads measured in early stages of the disease are associated with increased mortality [13]. Both SARS-CoV and MERS-CoV are able to evade the host's initial immune response by interfering with the synthesis of interferon (INF) I and by inducing apoptosis of T lymphocytes [14,15]. T lymphocytes play a fundamental role in generating an immune response to the virus and in modulating the inflammatory response of monocytes-macrophages [16]. Studies in animal models of SARS-CoV infection have shown that intense virus replication associated with reduced production of INF I during early stages of infection are associated with subsequent development of lung damage and with increased lethality [15]. The host's delayed immune response associated with increased viral replication promotes a progressive accumulation of inflammatory cells such as monocyte-macrophages, dendritic cells and neutrophils [17] in the lung tissue. When these cells are not appropriately modulated, the “immune” phase of the disease is triggered and a “cytokine storm” is generated [18].

Individual factors play a key role in the natural history of COVID-19. As already observed in SARS [19] and MERS [20], the most serious and lethal forms of COVID-19 are much more frequent in elderly patients, in males, and in individuals already affected by other pathologies. Epidemiological data collected in Italy at the time of this article shows that 95% of all deceased COVID-19 patients were ≥60 years of age and that 65.3% were males. Out of 1738 deceased patients for whom it was possible to examine clinical data, 82% had two or more pathologies, and the number of those suffering from a single pathology was four times the number of those with no pre-existing pathologies [21]. A recent meta-analysis of thirteen retrospective studies conducted in China (on a total of 3027 patients) has shown that age >65 years is a risk factor for the development of severe forms / mortality from COVID-19 with an odds ratio (OR) of 6.06; according to the same study, male sex results in a risk factor with an OR of 1.76, while the following pathologies constitute independent risk factors for severe forms/ mortality from COVID: hypertension (OR = 2.72), diabetes (OD = 3.68), cardiovascular disease (OR = 5.19), respiratory disease (OR = 5.15) [22]. Another meta-analysis of six studies conducted in China (on a total of 1558 patients) confirmed that the previous pathologies, as well as cerebrovascular disease (OR = 3.89), constitute independent risk factors for severe forms / mortality from COVID [23].

On the basis of these data, we have developed an algorithm to identify individuals at risk of developing severe or lethal forms of COVID-19. The algorithm uses three parameters: age, sex and comorbidity. Risk factors are: age ≥65 years, male sex, and the presence of at least one of the following pathologies: hypertension, diabetes, respiratory diseases, cardiovascular disease, cerebrovascular disease. Subjects with at least two of these three factors are considered at risk (**Table 1**).

**Table 1:** Algorithm for the identification of subjects at high risk of developing severe forms of COVID-19

Parameters	Risk factors
Age	≥65 years
Sex	Male
Comorbidity	1 or more diseases*
Risk assessment	1 out of 3 (low risk patient)
	≥2 out of 3 (high risk patient)

*Hypertension, diabetes, cardiovascular disease, respiratory diseases, cerebrovascular disease [22,23].

Individuals at risk could be sorted from national or regional health databases through simple search filters. General practitioners working in the first line of home intervention could thus identify subjects at risk among their patients. General practitioners could then verify the health conditions of patients, instruct them on COVID-19 symptoms (fever, cold, anosmia, sore throat, cough, etc.) and evaluate them in order to establish their suitability for antiviral therapy (eg, considering interference with other drugs, contraindications, etc.).

There are hundreds of clinical trials for SARS-CoV-2 therapies currently under way, and many involve drugs previously used against SARS-CoV and MERS-CoV [24]. Preliminary studies conducted on patients with COVID-19 show that early treatment with antivirals can reduce patient mortality, while late-stage treatment does not improve survival [8,9]. The biphasic nature of COVID-19, as already shown in SARS [6,7], could explain why treatments with antiviral drugs are useful just during the first phase of the disease.

Because of the current pandemic crisis, while awaiting the first results of trials, several antiviral treatments are being administered to patients with moderate and severe forms of COVID-19 [25]. A few days may elapse between the onset of symptoms and the clinical worsening of the disease; according to reported data, hospitalization takes place, on average, seven days after the onset of symptoms [4]. Therefore, most patients begin antiviral therapy only within hospitals. The delay between the onset of symptoms and the administration of antiviral drugs could reduce the effectiveness of these treatments [8]. For individuals at risk, treatment with antivirals should therefore start at home as early as possible after the onset of symptoms. Ideally, high-risk patients should start treatment immediately after disease is confirmed by nasopharyngeal swabs. However, if it is expected that the result of the swab cannot be available within 48-72 hours from the onset of symptoms, it may be ethically correct to start treatment earlier in highly suspicious cases: patients at risk who came into contact with COVID-19 patients in the previous 14 days and patients at risk with flu-like symptoms but previously vaccinated for influenza. In these cases, whether or not to continue with therapy would depend on results of nasopharyngeal swabs and / or on the evolution of patients' clinical conditions.

**Figure:**
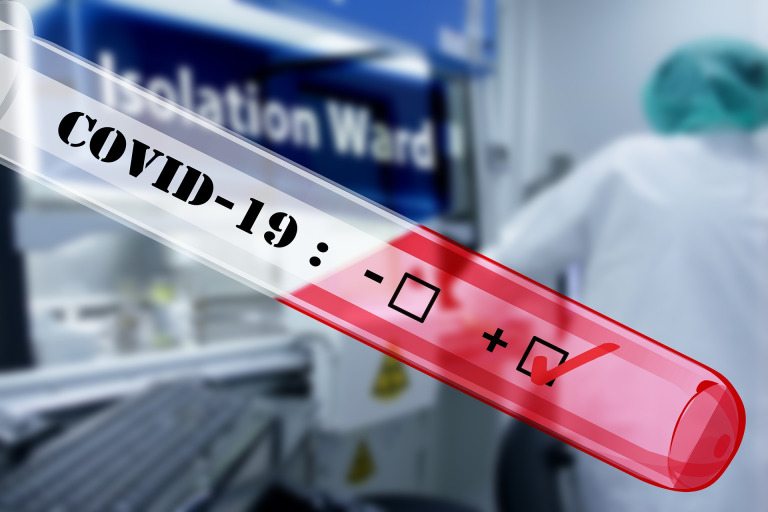
Photo: Image by Gerd Altmann from Pixabay.

Antiviral drugs used for early home treatment of patients at risk should meet the following requirements: a) must have already shown to have antiviral activity against the most pathogenic CoVs; b) must be already on the market and immediately available to the whole population; c) must have a good safety profile; and d) must be suitable for home use.

To date, only a few drugs are potential candidates for meeting all the requirements above: Chloroquine (CH), its analogue Hydroxychloroquine (HCH), and the combination Lopinavir (LPV) / Ritonavir (RTV). These drugs have been shown to have good antiviral activity on CoVs, especially during in vitro studies [7,26-29]. Unfortunately, there are only a few clinical studies [8,30,31], which – although encouraging – provide a sufficient grade of recommendation only if considered within the context of a serious emergency such as the current pandemic. A multinational hospital registry analysis recently highlighted the potential cardiologic risks of CH/HCH used to treat hospitalized COVID-19 patients [32]. In order to mitigate risks for patients, early home therapy would be conditional to a clinical evaluation of possible effects on pre-existing pathologies and of possible interactions with other drugs. For patients with macular degeneration, for example, it would be preferable to use LPV/RTV rather than CH/HCH even if the risk of worsening retinal disease in short-term treatments with HCH is low [33]. CH/HCH should not be prescribed at home if patients have QT prolongation or a predisposition to this condition [34]. Any other antiviral drug that will be proven to be effective for treatment of COVID-19, while maintaining the aforementioned characteristics of availability, safety, and ease of handling, could be considered for early treatment of patients at risk.

As with most strategies, starting early treatment for large numbers of patients with risk factors would imply some drawbacks. As mentioned above, risks from side effects could be minimized through clinical evaluation by general practitioners and by prescribing drugs with a long and well documented history of use. The potential benefits of early treatment for patients at risk of developing serious or lethal forms of COVID-19 should therefore exceed other clinical concerns. From an economic point of view, any reduction in the number of patients requiring intensive care would result in a significant advantage, since daily cost of intensive care with mechanical ventilation can exceed US$10 000 [35]. Even when prescribed to a large number of patients, the financial costs of therapies involving generic drugs like LPV/RTV or CH/HCH would be quite low. Implementation of the proposed strategy would also require a significant logistic effort, but coordinating general practitioners on a large scale and analyzing patient databases are activities of a magnitude comparable to those already being implemented to address the current pandemic in most countries.

In conclusion, COVID-19 is a pathology with important analogies to SARS and MERS. Previous studies on these two viruses are an important scientific legacy that could allow us to gain valuable time during a public health emergency. The subjects most at risk for COVID-19 are elderly males with pre-existing pathologies. Further studies will be needed to confirm the validity of the risk factors used in our algorithm. Both the cutoff used for age and the type and / or number of comorbidities could be modified if results from studies with larger samples or from other regions of the world become available. Early antiviral treatment of symptomatic patients at risk could result in a reduction in the numbers of hospitalizations and intensive care treatments and, therefore, of the related costs incurred by public health systems. In order to better manage the current COVID-19 pandemic, it would be appropriate to conduct a short-term formal assessment of a public health strategy based on the identification of subjects at risk and the treatment with antivirals during early stages of disease.
